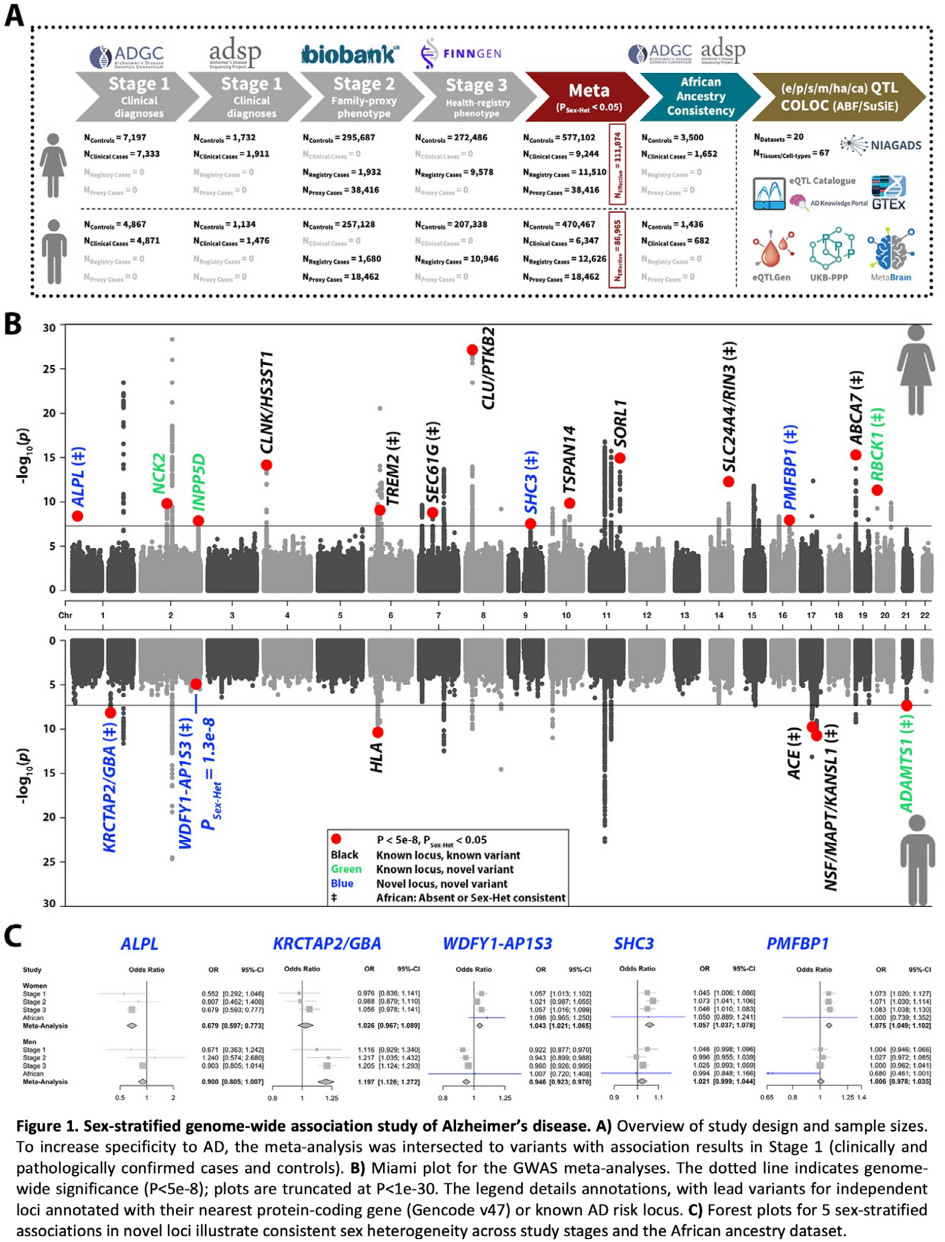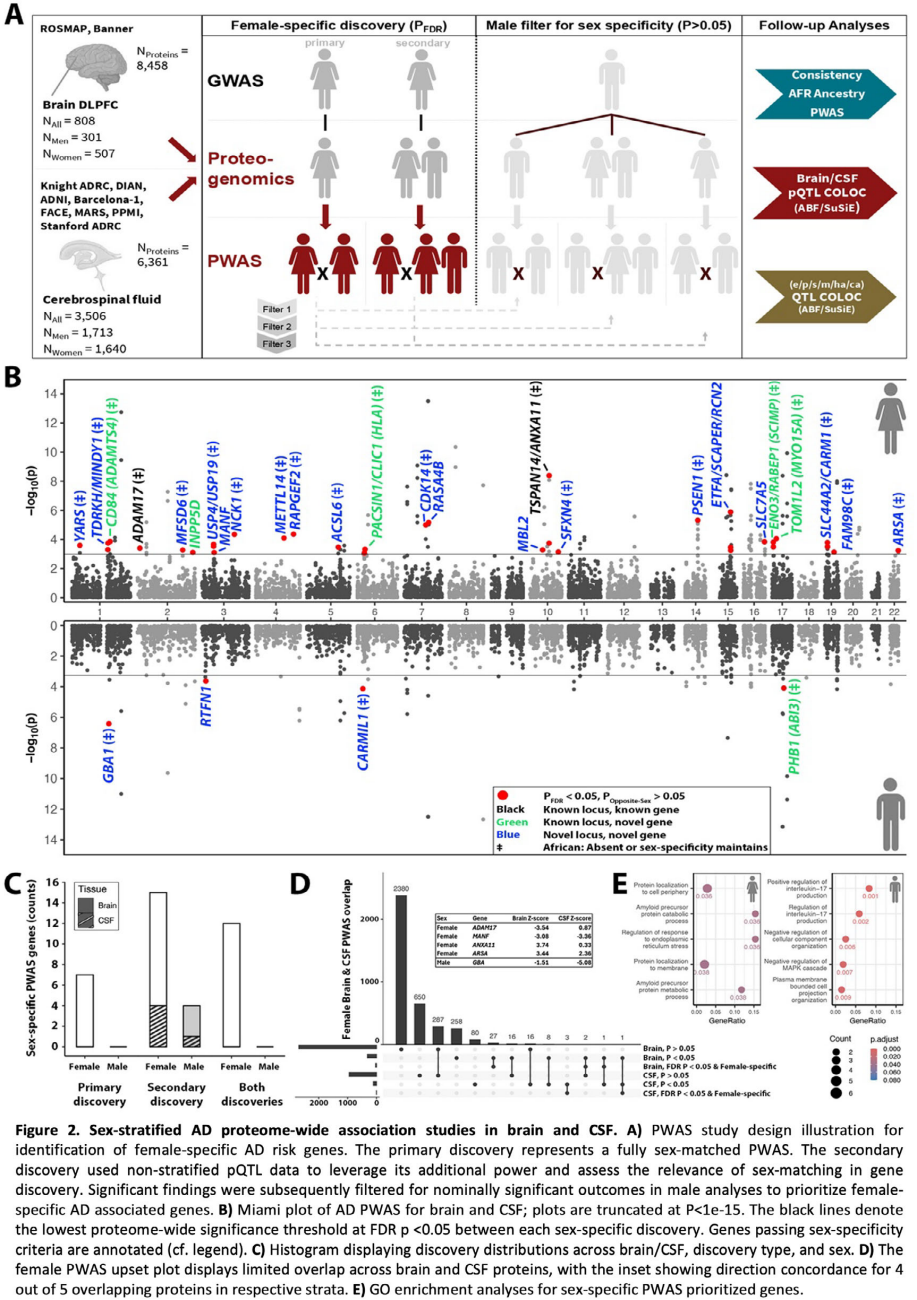# Cross‐ancestry prioritization of sex‐specific Alzheimer's disease risk genes through genetics and proteogenomics

**DOI:** 10.1002/alz70855_099094

**Published:** 2025-12-23

**Authors:** Danielle M. Reid, Noah Cook, Chenyu Yang, Soomin Song, Catherine Apio, Daniel Western, Yann Le Guen, Ilaria Stewart, Christina B. Young, Elizabeth C. Mormino, Valerio Napolioni, Zihuai He, Andre Altmann, Aliza P. Wingo, Thomas S. Wingo, Carlos Cruchaga, Yun Ju Sung, Michael D. Greicius, Michael E. Belloy, FinnGen

**Affiliations:** ^1^ NeuroGenomics and Informatics Center, Washington University School of Medicine, St Louis, MO, USA; ^2^ Department of Neurology, Washington University School of Medicine, St Louis, MO, USA; ^3^ Department of Psychiatry, Washington University School of Medicine, St. Louis, MO, USA; ^4^ NeuroGenomics and Informatics Center, Washington University School of Medicine, St. Louis, MO, USA; ^5^ Department of Psychiatry, Washington University School of Medicine, St Louis, MO, USA; ^6^ Department of Neurology and Neurological Sciences, Stanford University School of Medicine, Stanford, CA, USA; ^7^ School of Biosciences and Veterinary Medicine, University of Camerino, Camerino, Macerata, Italy; ^8^ Quantitative Sciences Unit, Department of Medicine, Stanford University, Standford, CA, USA; ^9^ The UCL Hawkes Institute, University College London, London, England, United Kingdom; ^10^ Department of Psychiatry, University of California, Davis, Sacremento, CA, USA; ^11^ Veterans Affairs Northern California Health Care System, Sacremento, CA, USA; ^12^ Department of Neurology, University of California, Davis, Sacremento, CA, USA; ^13^ Washington University School of Medicine, St. Louis, MO, USA

## Abstract

**Background:**

To elucidate sex differences in Alzheimer's disease (AD), we conducted the largest‐to‐date sex‐stratified genome‐wide association study (GWAS) of AD. To further increase power and identify sex‐specific, potentially druggable AD causal proteins, we performed proteome‐wide association studies (PWAS) integrating GWAS with proteogenomic (i.e., protein quantitative trait locus [pQTL]) brain and cerebrospinal fluid (CSF) datasets.

**Method:**

Sex‐stratified and sex‐heterogeneity AD GWAS were conducted in European ancestry individuals using a 3‐stage design, followed by fixed‐effects meta‐analysis (Figure 1A). PWAS were conducted via FUSION, combining sex‐stratified AD GWAS with sex‐matched and non‐sex‐stratified protein‐specific variant weights, respectively (Figure 2A). Significant findings in European ancestry were evaluated for sex heterogeneity consistency with admixed African ancestry AD GWAS (improved sex heterogeneity *p*‐value upon fixed‐effects meta‐analysis) and PWAS (sample‐size weighted Z‐score combination improved in matching sex and non‐significant Z‐score [P>0.05] in opposite sex).

**Result:**

GWAS identified 1 sex‐heterogeneous, 14 female‐specific, and 5 male‐specific loci, with 13 out 20 total loci (65%) showing consistent sex heterogeneity in African ancestry data, and 5 out of 20 being novel AD risk loci (Figure 1B‐C). Brain and CSF AD PWAS identified 66 and 19 genes significantly associated with AD in females, respectively, whereas 23 and 17 were identified for males. Upon filtering for sex‐specificity, 34 (52%) female and 4 (21%) male‐specific genes were identified (Figure 2B). Out of 38 total sex‐specific genes, 27 were present in 22 novel AD loci, and 23 out of 30 total unique loci (77%) showed persistent sex heterogeneity upon integration with African ancestry data (Figure 2B). The brain contributed 33 genes, of which 7 were uniquely observed through sex‐matched PWAS (Figure 2C). There were few overlapping significant sex‐stratified proteins between brain and CSF; however, 4 out of 5 overlapping proteins displayed concordant effect directions (Figure 2D).

**Conclusion:**

Sex‐stratified GWAS and PWAS identified 20 and 30 sex‐specific AD loci/genes, respectively, with high sex heterogeneity accordance in exploratory African‐admixed AD GWAS and PWAS. To provide validation and help prioritize probable causal genes at novel, significant GWAS/PWAS loci, colocalization analyses were performed with various QTL datasets (data not shown). These findings enhance our understanding of AD pathogenesis and risk, which may inform drug target development relevant to sex‐specific personalized medicine.